# Item generation for a proxy health related quality of life measure in very young children

**DOI:** 10.1186/s12955-020-1271-1

**Published:** 2020-01-14

**Authors:** Janine Verstraete, Lebogang Ramma, Jennifer Jelsma

**Affiliations:** 1 Faculty of Health and Rehabilitation Sciences, Division of Physiotherapy, Cape Town, South Africa; 2 Faculty of Health and Rehabilitation Sciences, Division of Communication Sciences and Disorders, Cape Town, South Africa

**Keywords:** Child, Infant, Toddler, Pre-schooler, Health, Health-related quality of life, HRQoL, Proxy

## Abstract

**Background and aims:**

Very young children have a relatively high prevalence of morbidity and mortality. Health care and supportive technology has improved but may require difficult choices and decisions regarding the allocation of these resources in this age group. Cost-effective analysis (CEA) can inform these decisions and thus measurement of Health-Related Quality of Life (HRQoL) is becoming increasingly important. However, the components of HRQoL are likely to be specific to infants and young children. This study aimed to develop a bank of items to inform the possible development of a new proxy report instrument.

**Methods:**

A review of the literature was done to define the concepts, generate items and identify measures that might be an appropriate starting point of reference. The items generated from the cognitive interviews and systematic review were subsequently pruned by experts in the field of HRQoL and paediatrics over two rounds of a Delphi study.

**Results:**

Based on the input from the different sources, the greatest need for a new HRQoL measure was in the 0–3-year age group. The item pool identified from the literature consisted of 36 items which was increased to 53 items after the cognitive interviews. The ranking of items from the first round of the Delphi study pruned this pool to 28 items for consideration. The experts further reduced this pool to 15 items for consideration in the second round. The experts also recommended that items could be merged due to their similar nature or construct. This process allowed for further reduction of items to 11 items which showed content validity and no redundancy.

**Conclusion:**

The need for an instrument to measure appropriate aspects of HRQoL in infants and young children became apparent as items included in existing measures did not cover the required spectrum. The identification of the final items was based on a sound conceptual model, acceptability to stakeholders and consideration of the observability of the item selected. The pruned item bank of 11 items needs to be subject to further testing with the target population to ensure validity and reliability before a new measure can be developed.

## Background

Young children are more susceptible to illness with mortality in children under five years of age accounting for nearly 20% of the overall mortality rate globally [1, 2]. Technologies which decrease mortality and morbidity are now more widely available, albeit at a large cost (e.g. neonatal intensive care support, management of terminal diseases). Difficult decisions regarding resource allocation care thus often need to be made at both a health authority and individual patient level and cost-effectiveness analysis (CEA) can guide these decisions [3]. To assist decision makers, a common metric has been developed for CEA, the Quality Adjusted Life Year or QALY, which incorporates time spent in a health condition and Health-Related Quality of Life (HRQoL) [3]. There is a plethora of HRQoL measures in use, but most target adult and older children and it is unclear how appropriate the included dimensions and items are for younger children.

HRQoL is based on a multidimensional measurement approach [4]. This multidimensionality is subsequent to the World Health Organisation’s (WHO) definition of health which includes three dimensions of health: physical, mental and social [5]. The three dimensions of health and an additional dimension of functional status have been combined to form the four, generally accepted, dimensions of HRQoL: disease and symptoms thereof, health status, psychological and social functioning [4]. These dimensions are universal across the lifespan. Most definitions of HRQoL place importance on the perceived effects of health on physical, social/role, psychological/emotional, and cognitive functioning. Disease symptoms, perceptions of health, and overall Quality of Life are often included within the dimensions of a HRQoL measure [6]. HRQoL can be regarded as the perceived effect which a medical condition or its management has on a person which can be either general or specific to the health condition.

HRQoL measures can be divided into two main categories: disease-specific and generic measures. Disease-specific measures are typically developed to measure the effects of a specific disease or condition on HRQoL [7].Disease-specific measures are argued to be more responsive in that they detect disease-specific clinical changes [8]. Disease-specific measures are however limited to evaluating HRQoL in the disease that they were developed for and are thus unable to provide comparative data across disease groups or between disease groups and the general population [8]. Generic health measures can be used to collect data from both healthy and ill individuals. Generic measures thus have a wider application and can be used in population health surveys, burden of disease studies, epidemiological studies, screening, describing health status, developing management plans for individual patients, informing clinical policy and resource allocation decisions [8–14].

Measurement of HRQoL in very young children (defined here as under five years of age) is challenging as motor and cognitive development is rapid and measurement of HRQoL needs to take into account the changes which emerge with this development [15]. A further constraint is that young children are unlikely to self-report reliably and measures for very young children need to rely on proxy-report [16]. This has the disadvantage that several items in existing HRQoL measures require the proxy to report on the subjective experience, of the child which may reduce the reliability of the measure [16]. Thus the Food and Drug Administration (FDA) and International Society for Pharmacoeconomics and Outcomes Research (ISPOR) guidelines suggest that proxy-measures for HRQoL be based on observable measures to minimise bias [16, 17]. A measure based on observable behaviour requires that each item is assessed according to observable behaviour of the child without the respondent having to draw conclusions regarding the child’s experienced HRQoL based on their own subjective assessment [16].

Systematic literature review of generic HRQoL measures for children had concluded that no existing measures were based solely on observable behaviour and most of the measures had not been developed based on a conceptual model [6, 18, 19]. There was thus a need to develop a new HRQoL measure, for young children, to be completed by proxy. To ensure that a new measure would add value to the existing measures, it was necessary to identify which items were of importance in this rapidly developing age group and how to ensure that reporting on items would be based on observable behaviour.

We reviewed a wide range of theories and models to identify the most appropriate guiding framework for the development of the new measure. The Wilson and Cleary Model is the oldest and most cited model of generic HRQoL [20] and suggests that the values and preferences of an individual will affect their overall HRQoL [21]. The Wilson and Cleary Model [22], the disease specific Taylor Model [23], and the International Classification of Functioning, Disability and Health (ICF) which includes a model of functioning [24, 25] all recognise the impact of personal factors and the environment in an individual’s perception of their HRQoL [20, 21, 23, 25, 26]. These models are similar in that they take into account the importance that personal factors and the environment make in the relationship between these key areas [20, 21, 23, 25, 26], they all take into account the presence or absence of disease or a health condition [21, 23, 25, 26] and they all include aspects of physical functioning [21, 23, 25, 26]. However, none of them account for the unique aspect of development which is a key aspect in the very young child.

The rapid development and acquisition of skills during the first years [27–29] of life also needs to be considered when developing a new measure. The theories of child development foreground the importance of the first years of life in shaping future adults and these early stages are commonly disrupted by negative experiences related to poor health [30–36]. Both the Taylor and ICF models take development and change in functioning over time into account, an element that is clearly essential when dealing with infants that are rapidly developing social, motor and other skills. Bakas et al. [20], however, suggest that the ICF may be more applicable across age and cultural groups as it was designed to describe the health of individuals, families, communities and populations across cultures. The ICF model with consideration of child development across the age range was thus used to inform the choice of items in this study.

The aim of this study was to establish a bank of items which would cover all the components of HRQoL relevant to the age group. This was to be done by interrogating items in existing HRQoL measures for young children and by generating further items through engagement with stakeholders. The selection of items would take into account the ISPOR [16] and the FDA [17] guidelines for HRQoL instrument development for very young children. The process of generation of the item bank and descriptors included a systematic review of the literature of existing HRQoL for young children; cognitive interviews with stakeholders including the target group (caregivers of young children) and a Delphi study [37, 38] with experts in the field of child health and HRQoL to prune and finalise the item bank for further psychometric testing. The process and results of the literature review and cognitive interviews with carers are summarised below. This paper describes the Delphi study component in detail.

## Generation of comprehensive item bank

### Systematic review

A systematic review of HRQoL measures for children under seven years of age was undertaken both to identify suitable items and to identify an instrument which could be used as a departure point of discussion on HRQoL with caregivers.

### Methodology

#### Search strategy

A search strategy for use on electronic databases was developed based on previously published literature as well as expert knowledge from the task group. The only limit to the searches was the exclusion of non-English articles or abstracts. Articles were searched in each database from the beginning of each database until April 2017. Pearling, which entails using the literature at hand to identify additional relevant studies, was done by hand searching the references of sourced papers. The websites of identified measures were also consulted for additional manuals or reference papers. The following electronic databases were searched: PubMed, EMBASE, Web of Science, PEDro, EBSCOHost, Africa-wide, NiPAD, CINAHL, ERIC, Health Source- Nursing/academic edition, MEDLINE, PsycARTICLES and PsycINFO, Scopus, Academic Search Premier. Conference proceedings from ISPOR and ISOQoL for the years 2012, 2013 and 2014 were searched for relevant literature. The terms in the title (“Health-Related Quality of Life”, OR “Quality of Life” OR “well-being” OR “health status”) AND (“children” OR “paediatric” OR “pediatric” OR “infant*” OR “child*”) AND (“questionnaire” OR “instrument” OR “measur*”) AND “generic” AND (“validation” OR “develop*”) were used to identify articles. Self-report and proxy report generic measures of HRQoL, health status and wellbeing were included. Measures were excluded if: they were disease specific, their dimensions were restricted to demographic or environmental indicators, and they only measured a single dimension or were used exclusively in children over the age of seven years.

#### Data analysis

A data abstraction form was used to record the literature reviewed. The researchers referred to the COSMIN checklist and all the criteria were recorded as well as information pertaining to bibliographic details, description of instrument development, completion by self/proxy, descriptive dimensions, number of items, response options, reference of the question to the child’s normal behaviour or the behaviour of others, recall period and scoring of the instrument. Attention was paid as to whether the items related to observable behaviour, if this was defined and whether developmental changes were factored into the measures. A second reviewer corroborated on the analysis and conclusions.

##### Criteria for selection of HRQoL measure on which to model a new measure

- The dimensions (broad concept such as physical health or mobility) or items (specific concept such as walking, running, jumping) included on the measure should be observable as per ISPOR) [16] and FDA guidelines [17].
- The recall period should be short to eliminate recall bias [39, 40] as young children have increased lability due to their rapid development [41].
- Content validity needs to be sound and based on a transparent development process with a variety of stakeholders most especially including parents or children [37–39].
- The measure needs to have a scoring system [4, 7] preferably derived from IRT or Rasch Analysis [42–44] or preference based scoring [3, 12, 45].
- Sound psychometric properties in term of validity and reliability [46–53].
- Practicality in terms of cost of the instrument as well as personnel costs in terms of length of time to administer or complete the instrument which would be directly related to the number of items on the measure [4, 7, 8, 39, 46, 54].
- As the new measure will be developed in South Africa evidence of cultural validity of the instrument would be preferred.

## Results

**Fig. 1:**
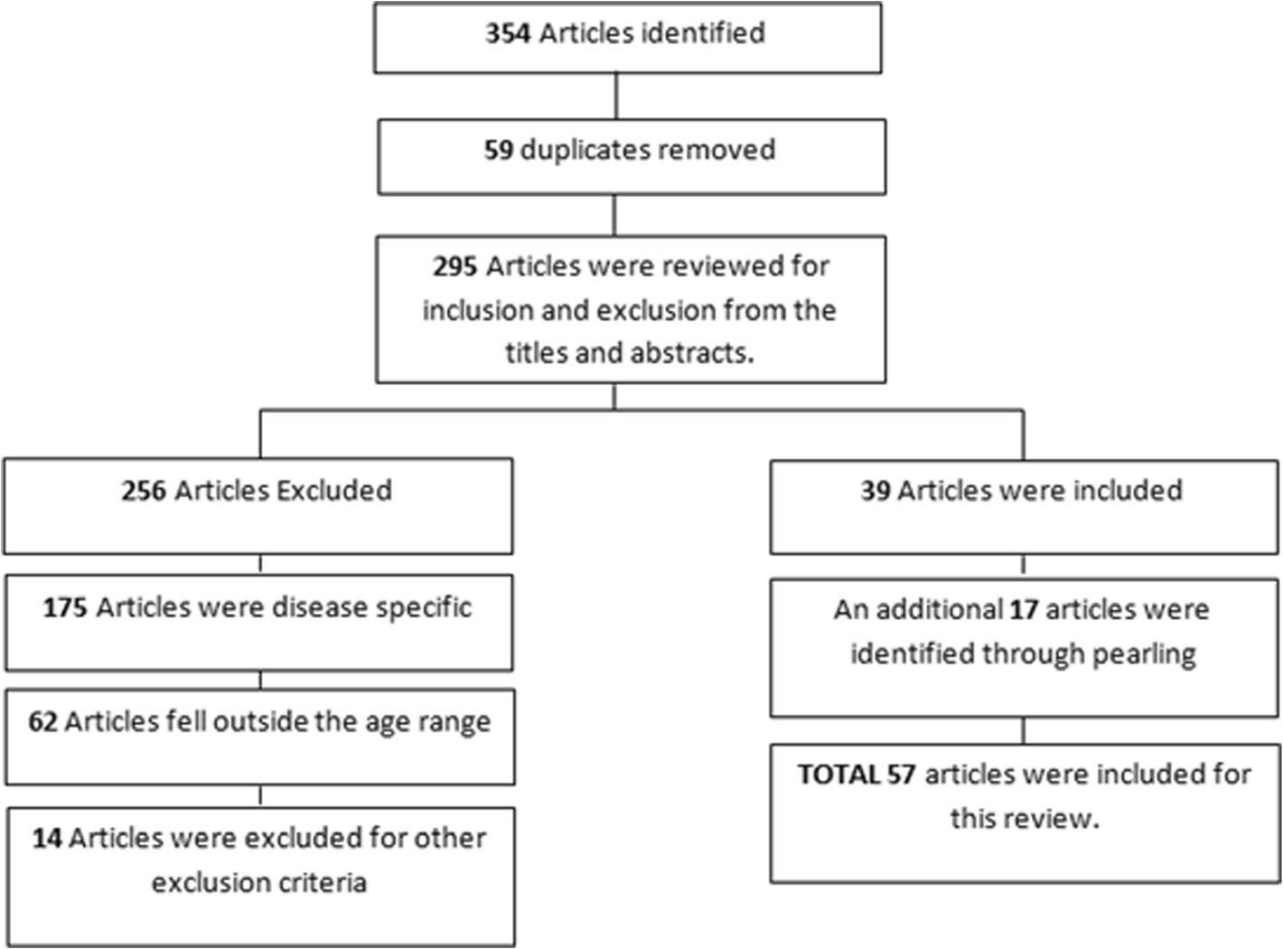
Search outcome for generic HRQoL measures

The 57 papers identified from the search (Fig. 1) resulted in 15 generic HRQoL measures to be reviewed. The measures included: Health Utilities Index (HUI); Health Status Classification for Pre-school children (HSCS-PS); Paediatric Quality of Life Inventory (PedsQL); The Warwick Child Health and Morbidity Profile (WCHMP), DISABKIDS Chronic Generic Module (DCGM); DISABKIDS Smiley Questionnaire (DSQ); The TZO-AZL Pre-school Children Quality of Life (TAPQoL); The Child Health Questionnaire (CHQ); The Infant and Toddler Quality of Life Questionnaire (ITQoL); The Kiddy-KINDL^R^; The Quality of Life Measure for Children (C-QoL); Patient Reported Outcome Measurement Information System – Paediatric Global Health (PROMIS- PGH-7); TEDQoL; Functional Status II R (FS II R) and the EQ-5D-Y Proxy.

## Summary/discussion

### Item generation

The items included in the existing measures were determined during the development stages mostly from the literature and from expert opinion. Parents were most often invited to comment on an item bank which subsequently guided inclusion of items. Items from each measure were determined from the literature or from a copy of the measure itself. Most of the measures have multiple items describing each respective dimension (HUI; PedsQL; DCGM; DSQ; TAPQoL; CHQ; ITQoL; Kiddy-KINDL^R^; C-QoL; TEDQoL; FS II(R)). Dimension inclusion was quite similar across measures with 15 measures including mobility/function, social dimension, cognition/learning, emotion and self-care included in eight measures. These dimensions were also generally the most observable dimensions on the measures. This was in keeping with the World Health Organisation’s (WHO) definition of health and components of the HRQoL namely: physical; emotional; social and cognitive function [4, 5]. The less observable dimensions of pain, behaviour, self-esteem and general health were not as well represented. Due to the similarity in items reported across measures all the dimensions found in the published literature were included in an item bank for further testing.

ISPOR guidelines suggest that to improve the objectivity in proxy reporting items should be based on behaviour that is observable to the proxy-respondent [16]. There is no measure that was based solely on observable behaviour. The DCGM, DSQ, WCHMP and PROMIS PGH-7 did not include any observability in their dimensions. The HSCS-PS, FS IIR, HUI and EQ-5D-Y had the highest number of observable dimensions. The PedsQL, TAPQoL, Kiddy-KINDL^R^ and CHQ all showed observability in at least half of their dimensions. Inclusion of observable dimensions would result in more accurate proxy report of HRQoL as it has been shown that observable dimensions such as physical activity correspond better between proxy and self-report than subjective outcomes such as emotion [55–57]. Ensuring observability with dimensions in children under four years of age has been recommended by both ISPOR [16] and the FDA [17]. These recommendations have been made in order to minimise subjectivity of the proxy reporter when completing proxy evaluation for very young children [16, 17]. This would in turn further improve the intra-rater reliability between two different proxy respondents.

### Selecting a measure on which to model a new HRQoL measure based on pre-set criteria

The results were similar to those found in previous reviews in that the development of few of the measures had been based on a conceptual/theoretical framework and that HRQoL or the construct of measurement was poorly defined [6, 58]. The HSCS-PS [53], PedsQL [59, 60]; PROMIS PGH-7 [43, 44, 61] and EQ-5D-Y Proxy [48, 62, 63] were identified as the most comprehensive generic HRQoL measures for proxy or self-completion in children under the age of seven years based on the pre-set criteria. However, each was not without its limitations. The observable characteristics of the dimensions were poorly defined in the literature and there was no measure that was based solely on observable behaviour. Most of the measures for children under the age of seven years were generic health profiles and had either dimension scoring and/or a single summary score. The HUI was the only preference based measure which allowed for utility generation, but this was based on the utility index from the adult general population [64, 65]. Most of the measures were developed and tested in High Income Countries such as the United States of America, Canada, Europe or Australia. Although this may be an artefact of inclusion of English articles or abstracts it does emphasis the Anglo-centricity of HRQoL measurement. As such if the measures are to be used in low income countries or ones that don’t have English as their main language, they need to undergo vast adaptations during validation process.

It emerged that there was a need for a new generic HRQoL instrument which would address the deficiencies noted. The need for a new measure was greatest for the very young child under three years of age. The review yielded a list of 36 candidate items from the existing measures. In addition, the EQ-5D-Y Proxy Version 1 for children aged 4–18 years (which elicits responses from the viewpoint of the proxy, rather than from that of the child) was identified as a useful departure point for discussion of HRQoL with the target population. It is short with five dimensions three of which are directly observable. The EQ-5D-Y development process included cognitive interviews with children and collaboration with experts in the field. Of importance it was, to the knowledge of the authors, the only measure to which South Africa researchers had contributed in the development stages. A latent value set has been developed for the EQ-5D-Y and utility weight generation is currently in progress [66, 67]. Although the validity and reliability of the proxy measure has only been reported in a Spanish study, it performed well in younger children [63]. Thus, the EQ-5D-Y was used as a point of departure introducing the concept of HRQoL in the cognitive interviews with caregivers.

## Cognitive interviews with caregivers of the target population

Cognitive debriefing with caregivers of the target population was used to further inform the development of the item bank. A descriptive, cross-sectional study design with data collected by means of an interviewer administered questionnaire was carried out. This section of the research explored the opinions of the caregivers regarding their child’s health, HRQoL and age.

## Methodology

The participants included caregivers of children aged from birth to seven years who were either acutely-ill, chronically-ill or from the general population. The inclusion of caregivers with children diagnosed with a spectrum of health classifications and across the age bracket was important to ensure that the new measure and included items were representative of the population for future use.

The caregiver of the child was defined as any person over the age of 18, who lived with the child and was wholly or partly responsible for the care of the child’s physical and emotional needs. Caregivers of children under seven years of age who were acutely-ill attending an acute health service (24 h or later post-admission), were chronically-ill attending out-patient clinics or members of the general population attending a day-care centre were included. As an item bank was being generated for an English HRQoL measure only English-speaking caregivers were recruited. There were 12 participants in each of the age categories divided across each of the institutions e.g. four caregivers of children aged 1–2 attending a day-care; four caregivers of children aged 1–2 who were acutely-ill; and four caregivers of children aged 1–2 who were chronically-ill.

### Instrumentation

The EQ-5D-Y Proxy version 1, which elicits a description of the child’s HRQoL from the proxy view point, was used to introduce the participants to the measurement of HRQoL. The dimensions included in the EQ-5D-Y include mobility, self-care, usual activities, pain or discomfort and worried, sad or unhappy. There is also a visual analogue scale ranging from 0 (worst health) to 100 (best health) [62]. After the completion of the EQ-5D-Y Proxy caregivers participated in a cognitive debriefing session guided by an interviewer-administered, self-designed questionnaire. The questionnaire comprised of both closed questions as well as open-ended questions which explored the reasoning behind the completion of the EQ-5D-Y Proxy. Participants were further asked to comment on the relevance of the dimension to their child and the use of wording and examples in the EQ-5D-Y Proxy. Caregivers were invited to suggest modification to the existing items as well as suggest any new items which should be added considering the age of their child. The face validity of the questionnaire was supported by two independent researchers.

The questionnaire was designed considering the verbal probing technique which asked the respondent specific questions. The questionnaire was constructed on an electronic mobile data collection platform, Magpi, which was used by the interviewer to guide the interview. The interviewer verbally asked each of the questions (some of which may have had a choice of two to three answers) and their answers to these questions were further ‘probed’ in order to identify the reasoning behind their response to the question [68]. Probing was done using scripted probes and were designed to elicit other items which the caregiver’s thought would be important to their child’s HRQoL and/or descriptions of these items for the child’s age. This method was selected due to the advantage that the interviewer was prepared for the interview and had control of the interview and the respondent did not require any training [68]. The verbal answers given by the respondents were manually recorded, by the interviewer, with the use of a tablet on the electronic Magpi system.

### Procedure

Ethical approval for the study was granted by the Human Research Ethics Committee of the University of Cape Town. Caregivers of 84 children were approached to complete cognitive interviews. The first four consecutive caregivers from each age category admitted to the in-patient facility, who attended the out-patient physiotherapy department, and in numerical order from the school list were recruited. Each participant completed the EQ-5D-Y Proxy Version 1 and then responded to the interviewer-administered questionnaire, which specifically probed the content of the existing instrument across age groups and the need for additional dimensions.

### Data analysis

The responses to the open ended questions were post-coded and inductive coding, often called ‘grounded’ coding was utilised as the codes were generated from the data [69]. Two researcher analysed the data for recurring themes, which were coded independently. Coding discrepancies were discussed until consensus was reached. In this way the narrative information was transformed into responses the frequency of which could be counted.

## Results

The layout, wording and time period for recall of the EQ-5D-Y were acceptable to the caregivers and considered appropriate for proxy completion. Furthermore, the VAS was generally well understood.

The caregivers identified items in addition to the five items included on the EQ-5D-Y. The items that could be added to increase the content validity of the new instrument according to age group presented in Table 1 below, in decreasing order of frequency.

**Table 1:** Proposed additional items

	Age Group (years)							
	0–1 (*n* = 12)	1–2 (*n* = 12)	2–3 (*n* = 12)	3–4 (*n* = 12)	4–5 (*n* = 12)	5–6 (*n* = 12)	6–7 (*n* = 12)	Total (*n* = 84)
Eating	9	8	5	4	6	4	1	37 (44%)
Communication	10	6	3	2	1		1	23 (27%)
Movement	12	2	1	2		2		19 (23%)
Toileting		2	4	3	3	4		16 (19%)
Sleep	7	2	2					11 (13%)
Play only	3	1	2			1	2	9 (11%)
Walking	2		3	2	2			9 (11%)
Discomfort		1	1	2	3		1	8 (10%)
Worried	1	1	1	1	1	1		6 (7%)
Independence			1	1	2			4 (5%
Upper limb movement	4							4 (5%
Cognition	1			2				3 (4%)
Emotion							2	2 (2%)
Kicking	2							2 (2%)
Motivation			1			1		2 (2%)
Self-esteem			1	1				2 (2%)
Sad		1				1		2 (2%)
Senses	2							2 (2%)
Socialize	2							2 (2%)
Crawling							1	1 (1%)
Dependence on Care	1							1 (1%)
Growth	1							1 (1%)
Hygiene	1							1 (1%)
Immunizations						1		1 (1%)
Perception					1			1 (1%)
Attitude					1			1 (1%)
Routine						1		1 (1%)
Sickness			1					1 (1%)
Unhappy	1							1 (1%)
Feeding		1						1 (1%)
Dexterity							1	1 (1%)
School Performance							1	1 (1%)
Achievement of Milestones	1							1 (1%)
Pride						1		1 (1%)

N = 84, * Caregivers did not all suggest additional items, and some suggested more than one item

The highest number of suggested additional items was in the younger age groups, most notably the 0–3-year group, with the 0–1-year group having the highest number of suggested additions. Communication and eating were identified as additional items which were of importance across all age groups. Communication was important for many caregivers as they felt that if their child was not physically able to participate or complete a task they were able to do this through communication with their family or peers. Eating was considered a fundamental attribute for health. Sleeping was suggested as a new item for children in the age band 0–3 years as caregivers felt that if children were unable to sleep well it would reduce their ability to play or learn. Toileting emerged as a new item for children over 1 year of age. The other suggestions related to existing dimensions but with change in nomenclature. Movement, as opposed to walking about, was suggested for the age group of 0–1 years. Play as an item on its own, as opposed to a list of descriptors under usual activities, was suggested across age groups as the descriptors of hobbies, sports and going to school were not deemed appropriate for younger children. Walking was suggested as an item due to the poor understanding of the term ‘walking about’. Discomfort and worried were suggested to be added as separate items. The dimension of self-care was suggested to be re-worded as assistance with self-care. All additional items were added for consideration during the Delphi rounds and these were either additions or reformulations of existing items (e.g. splitting up the PD dimension into two, pain and discomfort).

## Reduction of the item pool

The candidate item bank includes items identified in the literature review and cognitive interviews with caregivers of the target population. The reduction of items was determined through two rounds of Delphi panel deliberations.

## Method

### Study design and setting

A quantitative, consensus study was done with data collected by means of a two-part e-mail co-ordinated Delphi technique using the Content Validity Index (CVI) as a decision making tool [70]. The CVI is computed by dividing the number of experts giving a pre-determined high score by the total number of experts, this process ensures content validity for the items selected [70].

### Participants

Purposive sampling was used, and experts were selected based on their knowledge of either or both HRQoL and child health. Caregivers were not included in the exercise as their views had been explored cognitive interviews. The selected participants included international experts in HRQoL, child health and child advocacy. Professionals who practiced within a multi-disciplinary team and who had a vested interest in HRQoL and/or Health Economics and/or child development or who were active advocates for child health were included in the study (Table 2). Twelve of the 15 participants who were invited to participate in the study via e-mail invitation completed the first round. Subsequently seven experts participated in the second round. Due to anonymity in the completion of the Delphi study it is not known which of the experts participated at which stages.

**Table 2:** Details of participants invited to participate in the Delphi study

Number of Participants	Area of Expertise
6	Paediatricians with specialisations in: neurology, child development and education, rare diseases, drug advocacy, intensive care, palliative care and pain
5	Members of the allied paediatric health team including: physiotherapists, psychologists and a specialist professional nurse
2	Health economists
2	Public Health specialist

### Instrumentation

The design of the Delphi Questionnaire was based on templates which were available in the Survey Monkey survey management programme [71]. Participants were asked to rate the list of items generated through the literature review and the interviews with the care-givers using the CVI from 1 to 4 for each of the age groups. The CVI ratings scale were characterised as: 1- not relevant, 2- somewhat relevant, 3- quite relevant, and 4- highly relevant. The results from the cognitive interviews and cognitive interviews indicated a need for an instrument to measure HRQoL in children aged 0–3 years, thus items were pruned for this age group by the experts. The age groups included for the rating exercise included: 0–12 months (including children before their first birthday); 12–24 months (including children from the day of their first birthday to before their second birthday) and 24–36 months (including children from the day of their second birthday to before their third birthday). The sub-analysis by age band was decided due to developmental cut-off points of motor and language acquisition. Where most children start walking between their first and second birthdays from a gross motor perspective. Furthermore, from the age of two years children start to use simple phrases, start to follow simple commands and understand simple questions. Thus, it was important to assess whether items would be equivalent across the three age bands or if more than one questionnaire was needed to measure HRQoL in children between 0 and three years. The questionnaire was pre-tested by two independent researchers to approve the content, structure and comprehension of questions. Necessary changes were made according to their input.

### Procedure

After ethical approval was obtained participants were invited to participate in the item pool generation via e-mail. As HRQoL is a contested concept, the e-mail included an operational definition of HRQoL. The objectives and methods of the study were described and a link to the online survey management system of Survey Monkey [71] was included. Anonymity was insured as survey monkey allows for anonymous completion of their surveys. All information was gathered from participants using Survey Monkey software [71]. Willing participants were asked to give informed consent and to participate in the process within a three-week time period. Participants were then asked to rate each item on a CVI from 1 to 4 for each of the three age groups. The participants were given an opportunity to suggest new items to be added to the item pool. Participants were also asked to give their opinion on number of items to include in the questionnaire.

The participants who completed the first round of the study were invited to participate in the second round of the study and to complete the second round within a three-week period. The second round of the survey included the items with a CVI ≥ 0.78, from the first round of the study, for each age group. Participants were asked to rank the top seven items for each age group (a value of one reflecting the most important item) and to give the reasoning behind their answers. They were further asked to identify items which could be combined under a different title. The second round of the Delphi study took approximately 25 min. Participants were blinded to each other in both rounds of the study. The participants were allocated a number for summary reports as well as data analysis to ensure confidentiality.

### Statistical analysis

The CVI which has been used successfully by other instrument developers was chosen as a basis for final item selection [70]. The CVI from round one for each item was computed as the number of experts giving a rating of either three or four divided by the total number of experts, a method suggested by Polit and Beck [70]. The CVI was computed for each item per age band. The cut-off point of inclusion of an item was taken from Polit and Beck [70] with a recommendation of a CVI of ≥0.75 for 6–10 participants.

Additional participants were not recruited to take part in the second round of the Delphi study as the aim was to reach consensus amongst the same group of participants. Literature indicates that a minimum of three experts is needed to draw conclusions regarding content validity [70]. This may however have limited the interpretation of the results as the sampling of the experts in the final round was not known. The items used in the second round of the Delphi study were identified through the rating exercises of the participants in the first round. The items top scoring items for each age group were incorporated into the final item bank for further testing.

## Results

Local and international experts in the field were invited to participate in the Delphi Study. Out of the 15 experts who were invited to participate 12 gave informed consent and completed the first round of the online survey. Eight of the original 12 participants participated in the second round of the study. All eight participants completed the questions regarding the age group 0–12 months. Seven participants completed the questions regarding the age group 12–24 months. Six participants completed the questions regarding the age group 24–36 months.

A summary of the results from the two rounds for each category are depicted in Table 3. In the 0–12-month category, eating was ranked as the most important item, followed by pain. Apart from mood, the nine top ranked items received a CVI ≥0.63. In the 12–24-month category, play had the highest CVI, followed by eating and achievement of milestones. The top ranked 11 items received a CVI ≥0.43. Additional items to the younger age group identified for the 12–24-month age group include: behaviour, growth, communication, achievement of milestones and socializing. In the 24–36-month category, play, pain usual activities and mood were ranked the most highly. Sleeping, mood, behaviour and socializing were ranked in the top ten with majority of experts ranking them important.

**Table 3:** Top Ranked Items per age group after both Delphi rounds

	0–12 months	12–24 months	24–36 months
	CVI (*n* = 8)	CVI (*n* = 7)	CVI (*n* = 6)
Eating	0.88	0.43	
Pain	0.75	0.43	0.67
Play	0.75	0.76	0.67
Sleeping	0.75	0.57	0.5
Relationships	0.75	0.71	
Mood	0.5	0.57	0.5
Movement	0.63		
Sickness	0.75		
Growth		0.43	
Behaviour		0.43	0.5
Socializing		0.43	0.5
Communication		0.43	
Achievement of Milestones		0.71	
Usual Activities			0.67
Independence			0.67

The respondents had been requested to provide item descriptors based on observable behaviour as well as which items had similar constructs and could be grouped together for measurement. The results are summarised in Table 4 below.

**Table 4:** Top ranked items with expert opinion on item grouping and descriptors

Top ranked items	Expert opinion on factors to consider for item descriptors	Operationalised item for future testing^a^
Eating	Eating and growth could be combined to form one new item of eating. Descriptor suggestions for eating varied across the age group with the older age group again having a focus on independent feeding. Some of the descriptors suggested for the younger age group were applicable across the age group to indicate more about the health status of the child together with growth. These included the child’s ability to suck or chew and swallow as well as the absence of subsequent, gagging, reflux or aspiration. Another important indicator for health was suggested as the ability to feed comfortably without fatigue or fussiness*.*	
Growth		Eating *(adequate oral intake to sustain growth).*
Play	Usual activities for children in these age groups was play and thus the items could be merged. The repertoire of skills for play was directly dependent on age and the achievement of gross and fine motor skills as well as interaction with others. The interaction with others for play progresses from the caregiver initiating play in the youngest age group, to playing alongside other children for children aged 12–24 months to interactive play for children 24–36 months. Play was further described as being enjoyable and mostly involving objects or toys.	Play *(Enjoys playing with objects or toys)*
Usual Activities		
Relationships	Socializing formed part of relationships and the two items could be merged. Descriptors of relationships included the response and reciprocal interaction between the very young child and their mother/significant carer. This later into the ability of the child to communicate basic needs to their carer and their ability to respond with affection to family and close friends. This bond with family and close friends was thought to strengthen as the child advances in age.	Relationships *(Interacts with family members in an age-appropriate manner)*
Socializing		
Behaviour	Inclusion of behaviour was only considered important after 12 months of age. Behaviour was thought to indicate health, absence of pain and happiness. Another element of behaviour was suggested as appropriate responses to people, environment and activities.	Behaviour *(Aware of different situations and able to respond appropriately to new places and people)*
Communication	Communication was described in terms of verbal and non-verbal communication. Descriptors included examples of communication as well as the ability to make one’s needs known to the family or the world. The descriptors suggested for children under 12 months were focused on some of the elements of communication with reciprocal interaction with individuals and the child’s subsequent enjoyment thereof. After 12 months the ability to (verbally) communicate needs to their carer became important. There was also an emergence of interaction with other children (socialising) but the emphasis remained on good interaction with family. After 24 months interaction with other children emerged to a stronger degree.	Communication *(0–6 months: cooing, squealing, eye contact, smiling) (7–12 months: ‘gaga’ uses gestures like pointing) (12–19 months: single words) (19–24 months: puts two words together)(25–36 months: starts telling stories)*
Independence	Independence was only ranked as important for children 24–36 months of age. Examples given for independence included self-care activities such as washing, dressing, toileting as well as becoming independent in a known environment.	Helping with daily activities *(Age appropriate assistance with washing, dressing and toileting)*
Mood	The importance of the dimension was justified in terms of happiness or unhappiness, sadness and crying. There was a suggested element of consolability or ability to control/regulate to these emotions or moods with regards to a child becoming irritable when tired or hungry, and judgement would need to be made when not irritable for these reasons. Mood or emotions seemed to further form the basis of interaction with both the caregiver and the environment. Behaviours of crying and smiling were suggested to be good descriptors for this dimension.	Controlling Emotions (settles easily with familiar people, touch or sound)
Movement	Age specific movement is one of the observable characteristics of milestone achievement in young children and thus the two items could be combined. The movement descriptors suggest free, smooth and functional movement of all four of the limbs. They are however age specific suggestions with specific limb movements or higher functioning movement for older children such as running and use of hands.	Movement *(0–1 month: grasping, sucking) (2–5 months: plays while on tummy) (6–7 months: sitting)(9–11 months: crawling and standing) (12–36 months: walking)*
Achievement of Milestones		
Pain	Pain is generally non-specific in younger children and the caregiver needs to determine whether the child is expressing distress due to pain or other issues such as hunger or tiredness. Pain could be judged in a child by the persistence of their crying, their interaction with the environment, facial grimacing or general discomfort. In the verbal child, it is usually easier to establish the presence of pain. Pain is also said to have emotional and physiological effects.	Pain *(painful behaviour includes: grimace, restless movement, inconsolable cry)*
Sleeping	Descriptors of sleep included the ability to fall asleep, the quality and duration of sleep according to age appropriate requirements.	Sleeping *(falls asleep easily, has restful uninterrupted sleep and enough sleep) (0–3 months: 16–20 h a day) (3–6 months 15–16 h a day) (6–12 months: 11–14 h a day) (12–36 months: 10–13 h a day)*
Sickness	Sickness was considered as a general descriptor for anything which may affect the health of a child or an indicator of general health. Thus sickness, regardless of magnitude, would in effect negatively affect the child’s overall HRQoL.	Visual Analogue Scale measuring general health from 0 to 100

^a^*All item descriptors were developed based on comment from experts as well as review of the literature*

All of these items will be considered for inclusion on the new HRQoL instrument and include: Eating, Play, Relationships, Behaviour, Communication, Independence, Mood, Movement, Pain, Sleeping and Sickness (general health). These candidate items were further mapped to International Classification of Functioning and Disability – Child and Youth (ICF-CY) categories to examine whether the proposed instrument would reflect the conceptual framework identified [72]. The item mapping showed that all the ICF Categories were represented except for the categories of Environment and Personal Factors. This finding was similar to results from mapping other HRQoL instruments to the ICF [25, 73, 74] .

The items generated from each of the stages is summarised in Table 5 below with the final items for inclusion indicated in the fourth column. The items were operationalised based on expert feedback on the suggested descriptors for each item and review of the literature.

**Table 5:** Summary of item generation

Systematic Review	Cognitive interviews	Delphi Round 1	FINAL ITEMS FROM DELPHI ROUND 2
Walking	Walking	Walking	
Mobility	Movement	Movement	Movement
	Achievement of Milestones	Achievement of Milestones	
Physical function			
	Upper Limb Movement	Upper Limb Movement	
	Kicking		
	Crawling		
Family Activities			
Family Cohesion			
Social			
Relationships		Relationships	Relationships
Doing things with family or friends	Socializing	Socializing	
Usual Activities	Usual Activities	Usual Activities	
Washing	Washing		
Dressing	Dressing		
Hobbies	Hobbies		
Sport			
Play	Play	Play	Play
School	School		
	Learning	Learning	
	School Performance		
Cognition	Cognition	Cognition	
	Perception		
Mental Health			
	Motivation		
Emotion	Emotion		
Behaviour		Behaviour	Behaviour
Worried	Worried	Worried	
Sad	Sad	Sad	
Unhappy	Unhappy	Unhappy	
	Routine	Routine	
Self-care			
Independence	Independence	Independence	Independence
	Dependence on Care		
Sleeping	Sleeping	Sleeping	Sleeping
Eating	Eating	Eating (able to take food orally)	Eating
	Feeding	Feeding (Ability of child to feed him/herself)	
	Growth	Growth	
Toileting	Toileting		
Pain	Pain	Pain	Pain
Discomfort	Discomfort	Discomfort	
Mood		Mood	Mood
Energy		Energy	
Self-Esteem	Self-Esteem		
General Health			
	Sickness	Sickness	Sickness (general health)
	Immunizations		
Dexterity	Dexterity		
Senses	Senses	Senses	
Communication	Communication	Communication	Communication
	Trust		
	Attitude		
	Hygiene		
	Pride		
		Crying	

## Discussion

The two rounds of the Delphi study pruned the item bank from 53 items after the literature review and cognitive interviews to 11 after the two rounds of the Delphi study. The four broad domains of HRQoL namely: Physical, Social and Mental and functional status were all covered in the items selected. The systematic review revealed considerable overlap in the items included. Furthermore, the items identified in the literature review were not all based on observable behaviour as recommended by the FDA [17] and ISPOR [16]. The recommended age range for completion of the measures is quite varied with the PedsQL [75] and HSCS-PS [53] having the closest age of administration to that of the items in the bank (0–3 years). The items of eating and sleeping could be included in broad definition of Physical Health and General Health on the PROMIS PGH-7 although not specified [43, 44, 61]. Play is listed as one of the examples in the Usual Activities dimension on the EQ-5D-Y Proxy [48, 62, 76] and may fall under the expansive question on the PedsQL “playing with other children”. Play in early childhood is associated with cognitive, linguistic, socio-emotional, problem-solving and identity development [77, 78]. Furthermore it is thought to assist in acquisition of skill (fine motor and gross motor), the development of social relationships as well as a form of recreation [24]. Thus, play would overlap with many items on the other instruments such as PedsQL items of sport, friends and school activities. The items on the HSCS-PS of dexterity, learning and remembering; thinking and problem solving and PROMIS item of fun with friends would also overlap with the construct of play.

Psychological development is intertwined with the development of communication, behaviour and relationships. In the infant and with some toddlers these items could act as an indicator for progress of psychological development [31, 79, 80]. The items of communication, behaviour, mood and relationships are interlinked with each other as well as dimensions on the other measures which include mental health, mood or feelings (afraid/scared, sad, angry, worry, emotion, worried, unhappy), behaviour, speech and cognition (school activities, learning and remembering and thinking and problem solving). Independence in young children could portray itself in many ways. However, the results of the cognitive interviews and Delphi study likened this to taking responsibility for tasks such as self-care and this item is thus likened to that of “looking after myself” on the EQ-5D-Y and self-care on the HSCS-PS. Pain is explicitly expressed in on the EQ-5D-Y and HSCS-PS but may be inferred or the cause of many of the problems experienced in the items of the other measures. Sickness was considered, by the experts, to be any form of illness or other contributing factor leading to poor health that would negatively impact on the HRQoL of the child. This item of sickness could thus be likened to general health question on the EQ-5D-Y and PROMIS and is included in the HSCS-PS in utilisation of health care and the PedsQL in the question related to missing school. Movement is included in all of the instruments as the ability to walk except for the PROMIS which asks only of Physical Health. The PedsQL additionally asks about the ability to run and participate in sport or activity.

The items are representative of the conceptual framework of the ICF-CY categories of Body, Structure and Function, Activities and Participation [81]. As the instrument aims to be a HRQoL rather than a general QoL instrument, it is may not be surprising that environmental factors are not represented. The inclusion of external factors, such as building accessibility and policy regarding health and wellness related issues may very likely influence HRQoL, much as the health condition might. However, it is rather the perceived impact of these components of the ICF on the HRQoL of the respondents, rather than the factors themselves that needs to be included. As personal factors are not codified in the ICF and generally include demographic details such as age and gender, these are also not represented here.

The limitations of this study include the selection of participants was limited to experts who were known to the research group which introduced a selection bias and further limited the results as experts from sectors such as social work and education were not invited to participate. This limitation was diminished by the fact that all the experts who participated work within a multidisciplinary team. Of note the developmental paediatrician works together with the education department in determining school readiness and placement of children.

## Conclusion

In conclusion, a bank of items was selected based on the findings from the literature reviews, cognitive interviews and Delphi study. This process ensured that the items for inclusion were developmentally appropriate for the age range of inclusion. The final items included: behaviour, communication, eating, independence, play, mood, movement, pain, relationships, sickness (general health) and sleep. These items were representative of the definition of HRQoL and encompassed broader dimensions of physical (eating, play, movement, pain, sickness and sleep), emotional (behaviour, communication, mood) and social (behaviour, communication, independence, relationships). These items are also representative of the dimensions of the ICF: body structure and function, activities and participation. The eleven items and their descriptors will need to undergo further testing with the target population for their feasibility and utility before the final measure is developed.

## Data Availability

The datasets used and/or analysed during the current study are available from the corresponding author on reasonable request.

## Abbreviations

CEA: Cost Effective Analysis
CHQ: The Child Health Questionnaire
C-QoL: The Quality of Life Measure for Children
DCGM: DISABKIDS Chronic Generic Module
DSQ: DISABKIDS Smiley Questionnaire
FDA: Food and Drug Administration
FS II R: Functional Status II R
HRQoL: Health Related Quality of Life
HSCS-PS: Health Status Classification for Pre-school children
HUI: Health Utilities Index
ISPOR: International Society for Pharmaco-economics and Outcomes Research
ITQoL: The Infant and Toddler Quality of Life Questionnaire
PedsQL: The Paediatric Quality of Life Inventory
PROMIS-PGH-7: Patient Reported Outcome Measurement Information System
QALYs: Quality Adjusted Life Years
QoL: Quality of Life
TAPQoL: The TZO-AZL Pre-school Children Quality of Life
VAS: Visual Analogue Scale
WCHMP: The Warwick Child Health and Morbidity Profile
